# Intranasal immunization with *Mycobacterium tuberculosis* Rv3615c induces sustained adaptive CD4^+^ T‐cell and antibody responses in the respiratory tract

**DOI:** 10.1111/jcmm.13965

**Published:** 2018-10-24

**Authors:** Jiangping Li, Jun Zhao, Juan Shen, Changyou Wu, Jie Liu

**Affiliations:** ^1^ Institute of Immunology Zhongshan School of Medicine Sun Yat‐Sen University Guangzhou China; ^2^ Laboratory of Infectious Diseases and Vaccine State Key Laboratory of Biotherapy West China Hospital Sichuan University Chengdu China

**Keywords:** adaptive immunity, CD4, intranasal, mucosal, *Mycobacterium tuberculosis*, Rv3615c, Th1

## Abstract

Sustained adaptive immunity to pathogens provides effective protection against infections, and effector cells located at the site of infection ensure rapid response to the challenge. Both are essential for the success of vaccine development. To explore new vaccination approach against *Mycobacterium tuberculosis* (*M.tb*) infection, we have shown that Rv3615c, identified as ESX‐1 substrate protein C of *M.tb* but not expressed in BCG, induced a dominant Th1‐type response of CD4^+^ T cells from patients with tuberculosis pleurisy, which suggests a potential candidate for vaccine development. But subcutaneous immunization with Rv3615c induced modest T‐cell responses systemically, and showed suboptimal protection against virulent *M.tb* challenge at the site of infection. Here, we use a mouse model to demonstrate that intranasal immunization with Rv3615c induces sustained capability of adaptive CD4^+^ T‐ and B‐cell responses in lung parenchyma and airway. Rv3615c contains a dominant epitope of mouse CD4^+^ T cells, Rv3615c_41‐50_, and elicits CD4^+^ T‐cell response with an effector–memory phenotype and multi‐Th1‐type cytokine coexpressions. Since T cells resident at mucosal tissue are potent at control of infection at early stage, our data show that intranasal immunization with Rv3615c promotes a sustained regional immunity to *M.tb*, and suggests a potency in control of *M.tb* infection. Our study warranties a further investigation of Rv3615c as a candidate for development of effective vaccination against *M.tb* infection.

## 1 INTRODUCTION

Tuberculosis (TB), caused by *Mycobacterium tuberculosis* (*M.tb*), is one of the most prevalent infectious diseases worldwide, accounting for 10.4 million new cases and 1.5 million mortalities annually.^1^ Vaccination with *Mycobacterium bovis* Bacillus Calmette‐Guérin (BCG) has made a marked contribution to the control of *M.tb* infection, especially in juvenile and newborns. However, BCG does not provide adequate protection for all age groups, particularly in adults.^2^ With the constant emergence of multidrug‐resistant strains, prevention of *M.tb* infection is the most promising and cost‐effective approach to reducing the TB epidemic.^3^ Therefore, there is an urgent need for the development of an effective vaccination strategy to protect against *M.tb* infections.

Vaccination primes antigen‐specific precursors, and induces their expansion and differentiation into memory cells. When these memory cells re‐encounter a cognate antigen, they mount a robust and rapid response to control the infection at early stage.^4^ In the case of a *M.tb* infection, there are more CD4^+^ T cells than CD8^+^ T cells at the sites of infection, and the CD4^+^ T cells have been shown to play multiple roles in initiating and propagating the T‐cell responses in animal models and human cases.^5^, ^6^ CD4^+^ T cells with effector or effector–memory phenotype played a major role in controlling the mycobacteria at site of infection and limited progression of the disease.^7^ Some of them had a phenotype of CD44^+^CD62L^low^,^8^ and produced Th1‐type cytokines, such as IFN‐γ, TNF‐α, and IL‐2. These effector cytokines eliminated the infected cells and controlled *M.tb* replication.^9^, ^10^ Thus, many vaccine developments have been focused on identifying new CD4^+^ T‐cell epitopes inducing Th1‐type responses, or modifying BCG to improve efficacy for providing a broader protection.^11^, ^12^ Among them, ESAT‐6 and CFP‐10, which induce dominant Th1‐type CD4^+^ T‐cell responses, have been evaluated and shown potentially protective effects. The ESAT‐6, formatted as an ESAT‐6‐Ag85 fusion protein, promoted strong and long‐lived *M.tb*‐specific T‐cell responses in naïve human volunteers^13^, ^14^; the CFP‐10, previously used for disease diagnosis, could induce IFN‐γ release and reduce IL‐4 secretion of CD4^+^ T cells, as well as promote antibody response with an enhanced IgG2a/IgG1 ratio in mice.^15^ Although the evaluation is ongoing, establishment of adaptive Th1‐type CD4^+^ T‐cell response with effector or effector–memory phenotype is one of the targets for vaccine development to prevent TB.

Induction of only Th1‐type memory responses did not sufficiently eliminate *M.tb*, nor did it efficiently protect the host; the location of vaccine‐primed memory cells also had an impact on the efficiency of protection.^16^, ^17^ Limited success of TB vaccine trials might simply be due to failure to elicit, maintain, and/or deliver sufficient effector–functional T cells to the respiratory tract.^18^, ^19^ A population of T cells that reside in peripheral tissues has been recently identified as tissue‐resident memory T cells (T_RM_).^20^, ^21^ They are at the frontline, encountering pathogen invasions, and are antigen‐experienced memory cells. They can expand rapidly in response to cognate antigens to establish a barrier that limits the infection of target cells without the time‐consuming procedure of recruiting effector cells from lymphoid tissues and the circulation.^22^ Intranasal immunization induces not only systemic but also airway mucosal immune responses, while subcutaneous immunization preferably elicits responses in the serum and peripheral lymphoid tissues.^23^ Intranasal immunization with BCG preferably induced TB‐specific CD4^+^ T cells in the lung tissue,^7^ and lung tissue CD4^+^ memory T cells‐mediated adaptive immunity against Bordetella pertussis and Coronavirus infections efficiently.^24^, ^25^, ^26^ The route of immunization has an impact on the location and differentiation of vaccination‐primed cells, and intranasal immunization has being tested as a promising approach to induce mucosal immunity in the respiratory tract for prevention of *M.tb*, influenza virus, respiratory syncytial virus, and other pathogenic infections.^11^, ^13^, ^26^, ^27^, ^28^

The escalating magnitude of T‐cell response and IFN‐γ production induced by BCG did not provide additional protection against *M.tb* infection.^18^ In search for new TB vaccine candidates, we evaluated Rv3615c, a protein whose secretion is dependent on a component of RD1, for potency of inducing T‐cell responses of patients with tuberculosis pleurisy.^9^ Rv3615c has previously been identified as an ESX‐1 substrate protein C (EspC) and has been known as a protein with similar amino acid length and homologous sequence as ESAT‐6, CFP‐10, and other members of the ESAT‐6 family.^29^, ^30^ The encoding region for Rv3615c is out of RD1 but its secretion is controlled by the ESAT‐6 secretion system.^31^ Although not expressed in BCG, Rv3615c is actively expressed and accessible to the antigen‐presenting process during intracellular *M.tb* infections in vivo.^32^, ^33^ In a mouse model, subcutaneous immunization with recombinant protein containing Rv3615c promoted Th1‐type cytokine productions in the spleen, and both CD4^+^ and CD8^+^ T cells were responsible for the elevated cytokine productions, and a portion of them coexpressed multiple cytokines.^34^ In human cases, Rv3615c or its overlapping peptides elicited PBMCs isolated from patients with active TB or latent TB infection (LTBI) to produce IFN‐γ, with a portion of them coproducing IL‐2.^35^ Rv3615 has been shown to contain multiple epitopes of human T cells, many of them induce predominately CD4^+^ T‐cell responses, with only a few of them inducing weak CD8^+^ T‐cell responses. Although the protection induced by subcutaneous immunization with Rv3615c was modest to virulent *M.tb* challenge, these data suggest the potential of Rv3615c as a vaccine candidate for inducing adaptive immunity beyond those elicited by BCG. Following previous studies, here, we use mouse model to explore if immunization with Rv3615c intranasally promotes sustained memory CD4^+^ T‐cell response in airway compartment locally, and to examine the profile of T‐cell response by comparing with those induced by subcutaneous immunization. Our study can provide information for rational design and inoculation route of a TB vaccine.

## 2 MATERIALS AND METHODS

### 2.1 Animals

Female C57BL/6 mice aged 6‐8 weeks were purchased from the Laboratory Animal Center of Sun Yat‐Sen University (S.C. XK 2016‐0029) and maintained under pathogen‐free conditions. Mice were age‐ and weight‐matched in each experiment. All animal studies were approved by the Zhongshan School of Experimental Animal Ethics Committee, Sun Yat‐Sen University, Guangzhou, China.

### 2.2 Antigen, adjuvant, and immunizations

**Figure 1:**
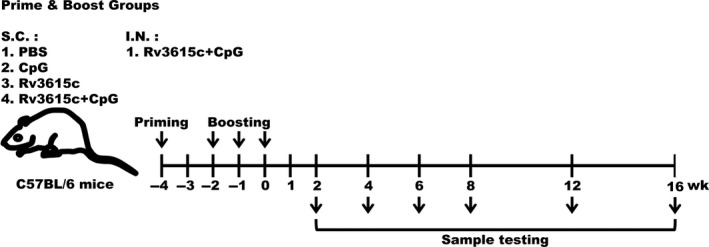
Scheme of immunization and sample test. C57BL/6 mice (n = 5‐9 per group) were subcutaneously (S.C.) or intranasally (I.N.) immunized following the prime‐boost protocol. Samples from lung, spleen, mesenteric, and inguinal lymph node (LN), bronchoalveolar lavage fluid (BALF), and serum were collected and tested at indicated time intervals

The *Mycobacterium tuberculosis* (*M.tb*) Rv3615c (Esx‐1 substrate protein C, EspC) protein and its peptide array, and CpG ODN 1826 were purchased from Sangon Biotech Co., Ltd (Shanghai, China) with customized synthesis. The peptide array comprises 19 peptides spanning the entire region of the Rv3615c sequence, with each having 15 mers and overlapping by 11 amino acids. The immunization protocol was previously reported.^36^ Briefly, 10 μg Rv3615c/mouse was mixed with or without 20 μg CpG ODN 1826/mouse. For subcutaneous immunization, the mixture was suspended in PBS and injected twice into the lower quadrant of the abdomen (200 μL/mouse). For intranasal immunization, the mixture was suspended in PBS and injected intranasally through the nasal cavity while the mice were anaesthetized with bromethane (20 μL/mouse). A detail schedule of prime and boost is shown in Figure 1.

### 2.3 In vivo labelling of cells in circulation

To distinguish cells in lung parenchyma (LP) from lung vasculature (LV), we used a protocol previously reported to label cells in circulation in vivo.^37^ In short, anti‐CD45‐phycoerythrin (clone 30‐F11, BD Bioscience) was diluted to 10 μg/mL in sterile PBS and injected into mice via the tail vein in a volume of 300 μL/mouse. The mice were killed 5 min later, and mononuclear cells were isolated from tissues for experiments. Cells labelled with anti‐CD45 were identified as in LV, and its counterpart was identified as in LP in flow cytometry data analysis.

### 2.4 Sample collection and cell preparation

Blood was obtained through retro‐orbital bleeding, and the serum was prepared following a standard procedure. Lung tissues were mechanically dissociated into 1‐2 mm^3^ pieces, then transferred to a culture dish containing 2 mL RPMI 1640, 5% fetal calf serum (FCS), 2 mg/mL collagenase IV, and 10 mg/mL DNase I (Sigma‐Aldrich, St. Louis, MO, USA), and incubated for 1 hour at 37°C in a shaker. Spleen, mesenteric, and inguinal lymph node (LN) tissues were mechanically disrupted and filtered through a 40 μm cell‐strainer (Falcon, Durham, NC, USA). Cell preparations from lung, spleen, and LN tissues were treated with red blood cell (RBC) lysis buffer, washed twice in complete RPMI 1640 medium, and loaded onto Ficoll‐Hypaque (Tianjin HaoYang Biological Manufacture, Tianjin, China) density gradient for isolation of mononuclear cells according to the manufacturer's instructions. The bronchoalveolar lavage fluids (BALF) were acquired by inflating lungs with 1 mL of complete RPMI 1640 medium via cannulation of the trachea and lavaging four times. Cells in the BALF were collected by centrifugation.

### 2.5 ELISA and ELISPOT assay

Cells were suspended in complete RPMI 1640 medium at a density of 2 × 10^6^/mL, and stimulated with or without protein or peptides (10 μg/mL) for 72 hours in a round‐bottomed 96‐well plate, 200 μL/well, at 37°C and 5% CO_2_. The culture supernatants were collected for detection of IFN‐γ, TNF‐α, and IL‐2 by enzyme‐linked immunosorbent assay (ELISA) according to the manufacturer's instructions (BD Biosciences, San Jose, CA, USA).

Cells were suspended in complete RPMI 1640 medium at a density of 2 × 10^6^/mL, and stimulated with or without protein or peptides (10 μg/mL) for 24 hours in precoated BD™ ELISPOT plates (BD Biosciences), 100 μL/well, at 37°C and 5% CO2. After washing, the plate was incubated with Biotinylated detection antibodies followed by Streptavidin‐HRP. AEC substrate was added into the wells for spot development, and the developing reaction was stopped by washing the wells with deionized water. The plate was air‐dried at room temperature, and the IFN‐γ‐producing cells were enumerated using ImmunoSpot S6 Analyser (Cellular Technology Ltd, Cleveland, OH, USA) according to the manufacturer's instructions. The result shown is the mean of triplicate well readings.

### 2.6 Flow cytometry analysis

Detection of cell phenotype and intracellular cytokine expressions using flow cytometry has been previously described.^9^ In short, for phenotyping, cells were washed with PBS containing 0.1% BSA and 0.05% sodium azide, and suspended into staining buffer. Cells were incubated with CD16/32 mAbs for 15 min on ice to block Fc receptors, then stained with phenotyping mAbs for 30 min at 4°C in the dark.

For intracellular cytokine staining, cells (2 × 10^6^/mL) were stimulated with or without protein or peptides (10 μg/mL) plus anti‐CD28 (1 μg/mL) for 12 hours at 37°C and 5% CO_2_. Brefeldin A (10 μg/mL, Sigma‐Aldrich) was added into the culture during the final 6 hours. The cells were washed twice with PBS containing 0.1% BSA and 0.05% sodium azide. After staining for phenotyping as described above, cells were fixed with 4% paraformaldehyde and permeabilized with 0.1% saponin in PBS with 0.1% BSA and 0.05% sodium azide overnight at 4°C. Cells were washed and stained with the indicated mAbs for intracellular cytokine expressions for 30 min at 4°C in the dark. Cell samples were assayed by FACS Aria II (Becton Dickinson), and data were analysed with FlowJo (TreeStar, San Carlos, CA, USA). The following antibodies/reagents were purchased from BD Biosciences and used for flow cytometry assay: anti‐mouse CD16/32 (clone 2.4G2); Phycoerythrin‐Texas Red (PE‐CF594) conjugated‐CD3 (clone 145‐2C11); Allophycocyanin‐Cyanin 7 (APC‐Cy7) conjugated‐CD4 (clone GK1.5); Phycoerythrin‐Cyanin 7 (PE‐Cy7) conjugated‐IFN‐γ (clone XMG1.2); Allophycocyanin (APC) conjugated‐IFN‐γ (XMG1.2); Fluorescein isothiocyanat (FITC) conjugated‐IFN‐γ (XMG1.2); Fluorescein isothiocyanat (FITC) conjugated‐TNF‐α (MP6‐XT22); Phycoerythrin (PE) conjugated‐IL‐2 (JES6‐5H4); Fluorescein isothiocyanat (FITC) conjugated‐CD44 (IM7); Allophycocyanin (APC) conjugated‐CD44 (IM7); Peridinin chlorophyll protein Cyanin 5.5 (Percp‐Cy5.5) conjugated‐CD62L (MEL‐14); Peridinin chlorophyll protein Cyanin 5.5 (Percp‐Cy5.5) conjugated‐CD103 (M290); Phycoerythrin (PE) conjugated‐CD45 (30‐F11); Allophycocyanin (APC) conjugated‐CXCR3 (CXCR3‐173); Phycoerythrin‐Cyanin 7 (PE‐Cy7) conjugated‐CXCR5 (2G8); Phycoerythrin (PE) conjugated‐CD127 (SB/199); Phycoerythrin‐Texas Red (PE‐CF594) conjugated‐CD279 (PD‐1) (J43); Phycoerythrin (PE) conjugated‐CD152 (CTLA‐4) (UC10‐4F10‐11); Fluorescein isothiocyanat (FITC) conjugated‐CD43 (S7); Phycoerythrin (PE) conjugated‐CD40L (MR1); and purified anti‐mouse CD28 (CD28.2). Fluorescein isothiocyanat (FITC) conjugated‐CD45 (clone I3/2.3) was purchased from BioLegend (San Diego, CA, USA).

### 2.7 Antibody detection

Detection of antigen‐specific antibodies in BALF and serum by ELISA has been previously described.^38^ In short, microtitre plates were coated with recombinant Rv3615c protein at 5 μg/mL in 0.1 M sodium carbonate buffer (pH = 9.6) overnight at 4°C. The plates were washed with 0.05% Tween 20 in PBS and blocked with 1% bovine serum albumin (BSA) in 0.05% Tween 20/PBS at room temperature for 2 hours. Serially diluted BALF and serum samples were added into the plates, followed by washing three times with 0.05% Tween 20 in PBS. The binding immunoglobulins (sIgA, IgA, IgG_1_, IgG_2a_, and IgM) were captured with horse radish peroxidase (HRP)‐conjugated goat anti‐mouse sIgA mAb (Elabscience, Wuhan, China), HRP‐conjugated goat anti‐mouse IgA mAb, HRP‐conjugated goat anti‐mouse IgG_1_ mAb, HRP‐conjugated goat anti‐mouse IgG_2a_ mAb (eBioscience, San Diego, CA, USA), and HRP‐conjugated goat anti‐mouse IgM mAb (Bethyl Laboratories, Montgomery, AL, USA) respectively. The plates were washed with 0.05% Tween 20/PBS and developed using TMB substrate. The reaction was stopped with 2N H_2_SO_4_, and data were collected by an ELISA reader. The end‐point titre was defined as the highest dilution that gave an absorbance value above the optical density of 0.1 and was analysed using GraphPad Prism 5 (GraphPad Software Inc., San Diego, CA, USA).

### 2.8 Statistical analysis

All statistical analysis was performed with GraphPad Prism 5 (GraphPad Software Inc.) by either unpaired Student's *t* test when comparing two groups, one‐way ANOVA for more than two groups, or two‐way ANOVA for two variables. Data were presented as mean or mean ± SD. ***P < 0.001, **P < 0.01, *P < 0.05.

## 3 RESULTS

### 3.1 Rv3615c induced long‐lasting adaptive CD4^+^ T‐cell responses to the cognate antigen

**Figure 2:**
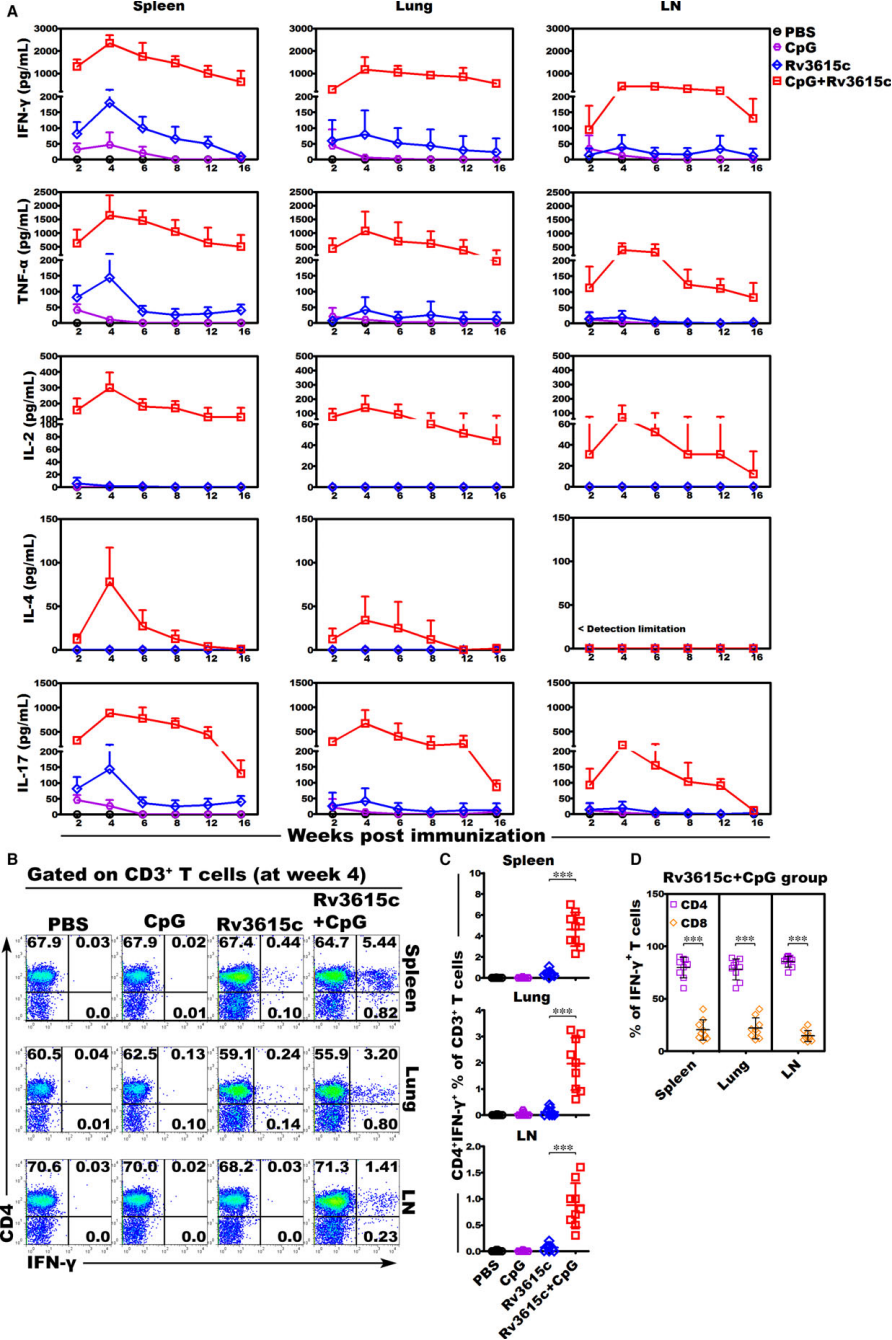
Immunization with Rv3615c induced sustained cytokine‐expressing CD4^+^ T cells systemically. Mice were primed and boosted S. C. with PBS, CpG, Rv3615c, and Rv3615c+CpG, respectively, following the immunization scheme. (A) At indicated time interval, mice were killed and mononuclear cells from spleen, lung and LN tissues were isolated and stimulated with Rv3615c. The supernatants were tested for cytokine productions by ELISA. (B‐D) Mice were killed at week 4 after boosting. Mononuclear cells from spleen, lung, and LN tissues were isolated and stimulated with Rv3615c. Brefeldin A was added into the culture during the final 6 h. The cells were harvested and stained with fluorochrome‐conjugated monoclonal antibodies for lineage differentiation and intracellular cytokine expression. Data were collected with flow cytometry and analysed with FlowJo. Representative dot plots in (B) showed the identification of IFN‐γ‐producing T cells. Frequency of IFN‐γ‐producing CD4^+^ T cell in immunization groups was shown in (C). Frequency of IFN‐γ‐producing cell in CD4^+^ and CD8^+^ population from Rv3615c+CpG group was shown in (D). Data shown were from nine mice and compared with Student's *t* test. Each dot represented individual mouse. ***P < 0.001

To explore the potency of adaptive immune responses induced by *M.tb* Rv3615c, we immunized mice subcutaneously (S.C.) with Rv3615c and Rv3615c plus CpG (Rv3615c+CpG) following the prime and boost scheme, respectively, and used PBS and CpG as controls (Figure 1). We examined mononuclear cells isolated from the spleen, lung, and mesenteric and inguinal LN tissues from the immunized mice for their cytokine production upon Rv3615c stimulation in vitro. Data showed that cells from mice immunized with Rv3615c+CpG produced higher level of cytokines than cells from those immunized with Rv3615c and controls (Figure 2A). Cytokine productions peaked at week 4, and maintained for at least 16 weeks, except for IL‐4 production, which diminished earlier than IFN‐γ, TNF‐α, IL‐2, and IL‐17, and dropped to baseline of controls at week 8. We also observed that IL‐17 production decreased significantly at week 12, while production of other Th1‐type cytokines maintained relatively steady. Mononuclear cells isolated from the LN had relative low cytokine production and retained the production for a shorter period than did cells isolated from other tissues, particularly for IL‐4 production. Data suggested that Rv3615c+CpG immunization established an adaptive immune response in our mouse model, and S.C. immunization induced a systemic immunity that was mostly predominated with Th1‐type responses. The Rv3615c+CpG ‐induced memory cells retained potency of responding to the cognate antigen at least 16 weeks. As an adjuvant, CpG enhanced the efficiency of Rv3615c immunization.

To find cell subsets responding to Rv365c, we analysed lineage differentiation of cytokine‐producing cells with flow cytometry. Data showed that most cytokine‐producing cells were CD3^+^ T cells, and Rv3615c+CpG was the most potent at inducing IFN‐γ production, with the frequency of IFN‐γ‐producing cells higher than that induced by other immunizations and controls (Figure 2B and C). This result was consistent with quantitative cytokine assay by ELISA. Among the cytokine‐producing T cells, majority (>80%) were CD4^+^CD8^−^ T cells, and less than 20% were CD4^−^CD8^+^ T cells (Figure 2D). Data suggested that Rv3615c preferably induced cytokine production of CD4^+^ T cells, consistent with our previous study on human PBMC.

### 3.2 Rv3615c contains an epitope of CD4^+^ T cells

**Figure 3:**
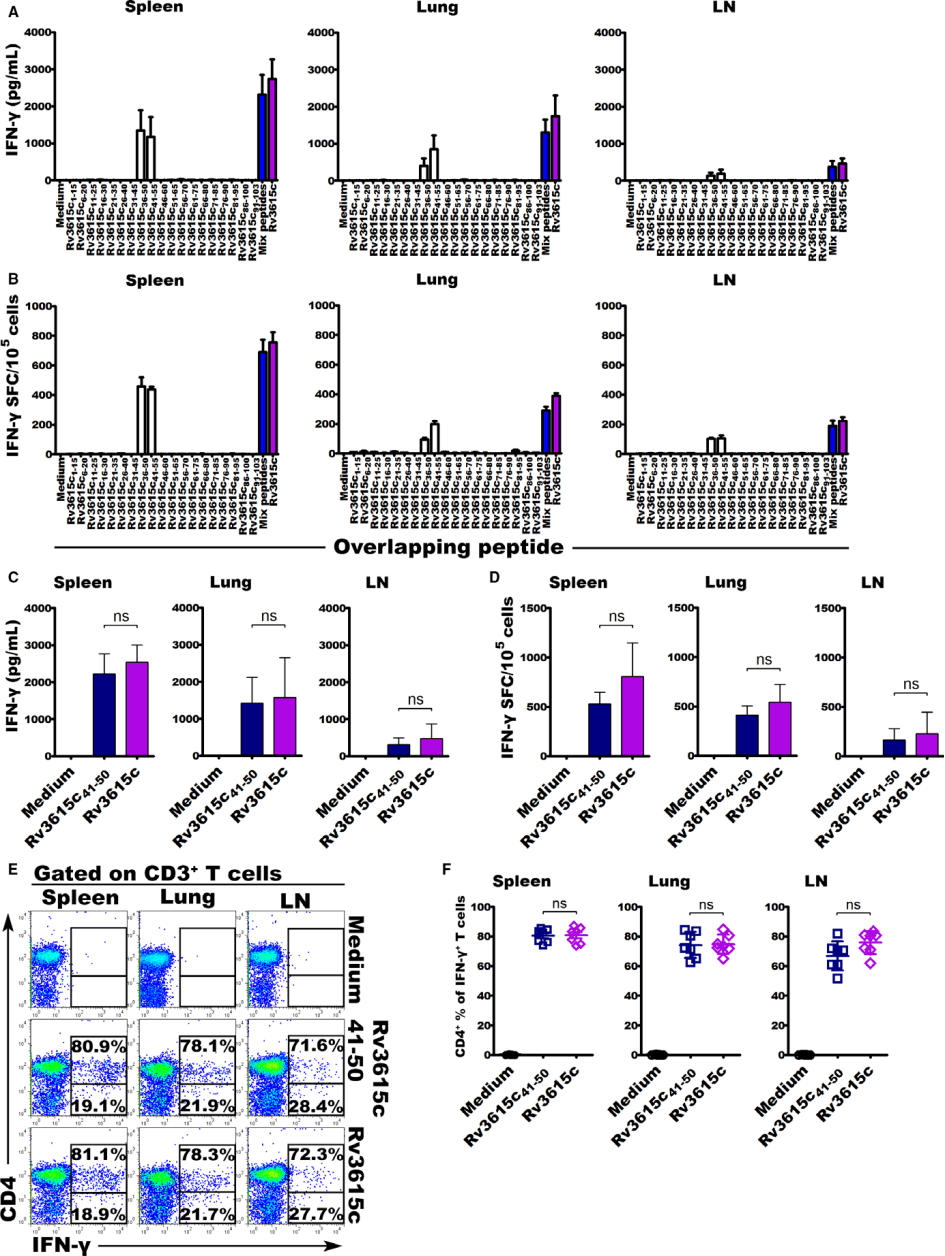
Overlapping peptide screening of epitope and its potential eliciting cytokine production. Mice were primed and boosted with Rv3615c+CpG S.C., and killed at week 4 after boosting. Mononuclear cells from spleen, lung, and LN tissues were isolated. (A & B) Cells were stimulated with individual overlapping peptides, mixture of the peptides and Rv3615c respectively. Medium for dissolving the peptides was used as controls. IFN‐γ production in the supernatant was assessed by ELISA, and cells producing IFN‐γ were counted by ELISPOT. (C & D) Cells were stimulated with Rv3615c_41‐50_ peptide, Rv3615c, and medium respectively. IFN‐γ production in the supernatant was assessed by ELISA, and cells producing IFN‐γ were counted by ELISPOT. (E & F) Cells were stimulated with Rv3615c_41‐50_, Rv3614c, and medium respectively. Brefeldin A was added into the culture during the final 6 h. Cells were harvested and stained with fluorochrome‐conjugated monoclonal antibodies and analysed for lineage differentiation and intracellular cytokine expression with flow cytometry. Representative dot plots in (E) showed the identification of IFN‐γ‐producing T cells by flow cytometry. Frequency of CD4^+^ cell in IFN‐γ‐producing populations was shown in (F), and compared with Student's *t* test. Data were from seven mice and expressed as mean ± SD with individual data point. ns: no significant difference

We used overlapping peptides to screen epitopes recognized by Rv3615c‐specific T cells. These peptides were 15‐mer each, overlapped by 10 amino acids and spanning the entire region of the Rv3615c sequence. Using quantitative ELISA and ELISPOT‐counting, we found that Rv3615c_36‐50_ and Rv3615c_41‐55_ elicited mononuclear cells isolated from Rv3615c+CpG immunized mice to produce IFN‐γ (Figure 3A and B). The magnitude of IFN‐γ production and the number of IFN‐γ‐producing cells almost reached the level of those elicited by full‐length Rv3615c and by mixture of the overlapping peptides, and were significantly higher than those elicited by medium control. We observed similar patterns of response in cells isolated from the spleen, lung, and LN tissues, with cells isolated from the LN showing relatively low IFN‐γ production, which was consistent with kinetic data showing in Figure 2A. Based on the screening, we proposed a T‐cell epitope within amino acid 41‐50, and generated peptide Rv3615c_41‐50_. We used Rv3615c_41‐50_ to stimulate mononuclear cells isolated from Rv3615c+CpG immunized mice and observed a magnitude of response similar to full‐length Rv3615c by quantitative IFN‐γ assessment and IFN‐γ‐producing cell‐counting (Figure 3C and D), which demonstrated a dominant T‐cell epitope at this location.

To study cell subset responding to Rv3615c_41‐50_, we analysed lineage differentiation of the IFN‐γ‐producing cells using flow cytometry and found that T cells producing IFN‐γ were mostly CD4^+^ (≥70%), and the frequencies were almost the same as those induced by full‐length Rv3615c (Figure 3E and F). Data demonstrated that Rv3615c contained an epitope of CD4^+^ T cells. This epitope was within amino acid 41‐50, and could induce a dominant response of CD4^+^ T cells specific to Rv3615c.

### 3.3 Intranasal immunization preferably establishes a long‐lasting adaptive immunity in BALF and lung

**Figure 4:**
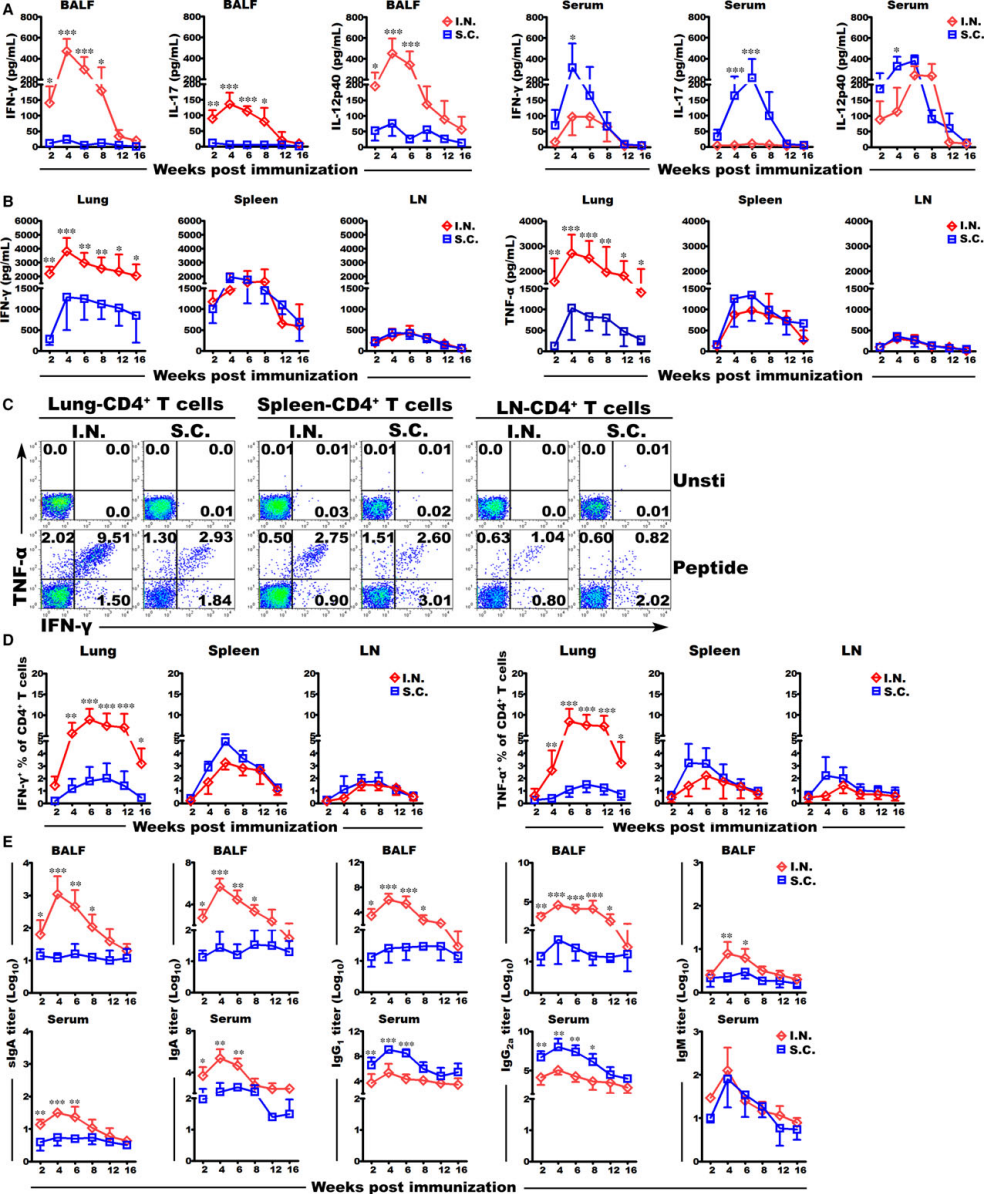
Intranasal immunization induced robust and sustained cytokines and antibodies in BALF and cytokine‐producing cells in lung tissues. Mice were primed and boosted with Rv3615c+CpG I.N. or S.C., and killed at the indicated time intervals. BALF, serum, lung, spleen, and LN samples were collected, and isolated mononuclear cells were suspended in complete RPMI 1640 medium. (A) BALF and serum samples were tested for IFN‐γ, IL‐17, and IL‐12p40 by ELISA. (B) Cells from lung, spleen and LN tissues were stimulated with Rv3615c_41‐50_, and the supernatant was tested for IFN‐γ and TNF‐α by ELISA. (C & D) Cells from lung, spleen and LN tissues were stimulated with Rv3615c_41‐50_. Brefeldin A was added into the cultures during the final 6 h. The cells were harvested and stained with fluorochrome‐conjugated monoclonal antibodies and analysed for lineage differentiation and intracellular cytokine expression with flow cytometry. Representative dot plots from flow cytometry analysis of samples 12 weeks after boosting were shown in (C), and frequency of IFN‐γ‐ or TNF‐α‐producing cells in CD4^+^ T population in lung, spleen, and LN tissues was shown in (D). (E) The BALF and serum samples were tested for Rv3615c‐specific sIgA, IgA, IgG_1_, IgG_2a_, and IgM by ELISA. Data shown were from seven mice and compared with Student's *t* test. Data were expressed as mean ± SD. *P < 0.05; **P < 0.01; ***P < 0.001

Since airway barrier is at the frontline against pathogen invasion, we wondered if intranasal (I.N.) immunization could preferably establish an adaptive immunity in the respiratory compartment locally. We immunized mice with R3615c+CpG I.N. following the prime and boost protocol, and compared cytokine levels in BALF and serum with samples from S.C. immunized mice from week 2‐16. We found that I.N. immunization induced higher IFN‐γ, IL‐17, and IL‐12p40 than S.C. immunization did in BALF (Figure 4A). The cytokine expressions peaked at week 4 after boost and maintained for at least 8‐12 weeks. In contrast, S.C. immunization preferably elevated cytokines in serum, and had little effect on cytokines in BALF. Next, we compared cytokine productivity of cells following I.N. and S.C. immunizations. We found that cells isolated from lungs of mice with I.N. immunization produced higher levels of IFN‐γ and TNF‐α upon Rv3615c_41‐50_ stimulation in vitro than did cells isolated from mice with S.C. immunization, while cells isolated from lymphoid tissues (spleen and LN) of mice immunized by those two approaches had the same IFN‐γ and TNF‐α production (Figure 4B). The increased cytokine production in airway and lungs by I.N. immunization peaked at week 4 after boost and maintained for at least 16 weeks.

Consistent with the quantitative cytokine assay, the frequency of cytokine‐producing cells preferably increased in the lungs following I.N. immunization. We stimulated mononuclear cells isolated from Rv3615c+CpG immunized mice with Rv3615c_41‐50_, and examinated the frequency of cytokine‐producing cells. Data showed that I.N. immunization induced a higher frequency of IFN‐γ‐ and TNF‐α‐producing cells in lung CD4^+^ T cells than S.C. immunization from week 2‐16 after boost, and these two immunizations induced the same frequency of cytokine‐producing cells in the spleen and LN tissues (Figure 4C and D). The kinetics of cytokine‐producing cells was consistent with patterns of quantitative cytokine assay after in vitro stimulation. Data suggested that I.N. immunization preferably established a long‐lasting adaptive cellular immunity in the respiratory tissues.

We also studied antibody responses to I.N. and S.C. immunizations in the airways and in serum. I.N. immunization induced a higher titre of Rv3615c‐specific antibodies than did S.C. immunization in BALF (Figure 4E). The isotypes of antibodies were secretary IgA (sIgA), IgA, IgG1, IgG2, and IgM. The antibody responses peaked at week 4 after boost, and maintained for about 8‐12 weeks, with the exception of IgM being maintained only for about 6 weeks. I.N. immunization also induced a higher titre of Rv3615c‐specific sIgA and IgA in serum than did S.C. immunization. The elevated sIgA and IgA in serum maintained for about 6 weeks. In contrast, S.C. immunization preferably induced IgG1 and IgG2 isotypes in serum, and induced the same titre of IgM as I.N. immunization did. Our data suggested that intranasal immunization preferably established a long‐lasting adaptive humoural immunity in respiratory system.

### 3.4 Intranasal immunization induced effector–memory CD4^+^ T cells with multicytokine coexpressions in lung

**Figure 5:**
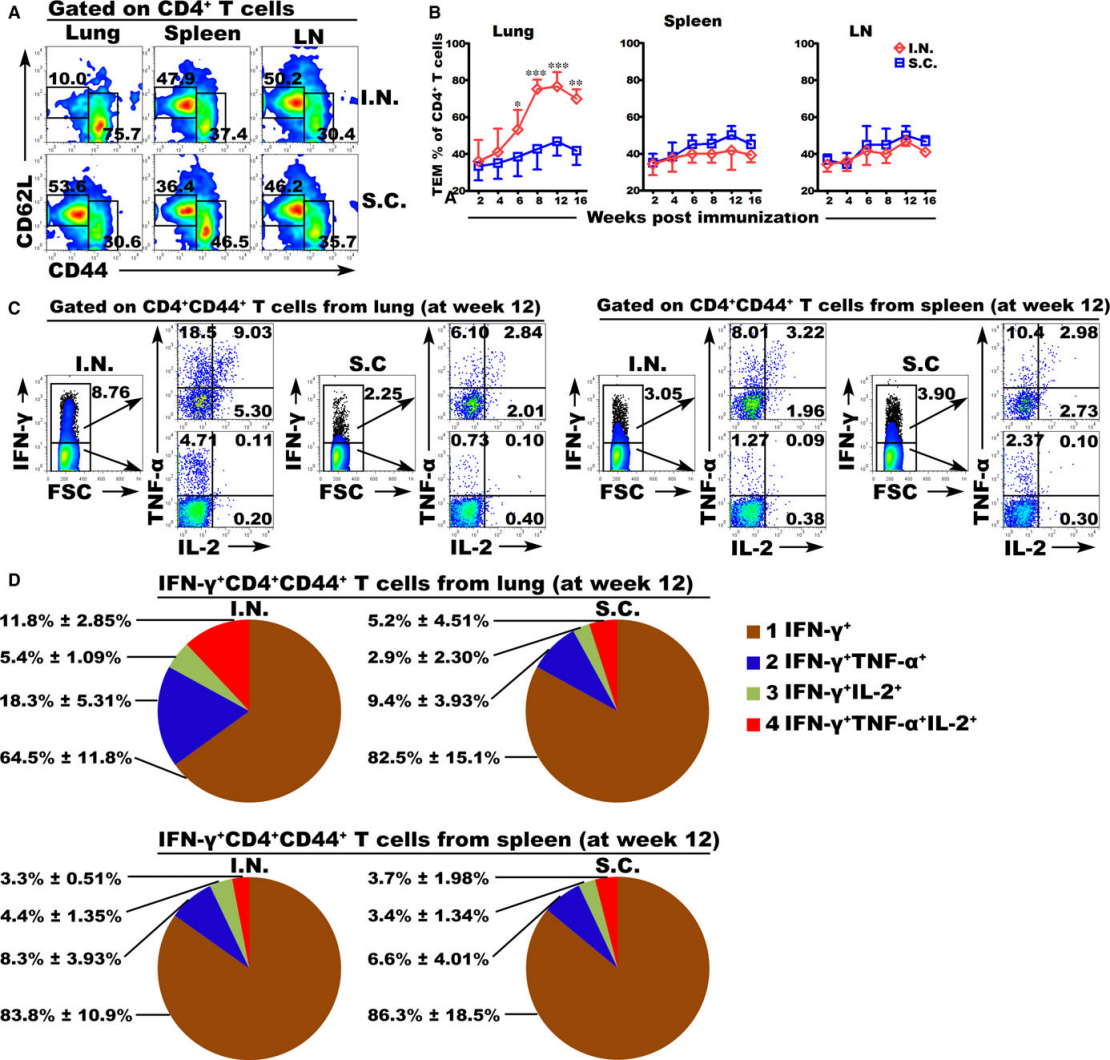
Intranasal immunization induced effector–memory CD4^+^ T cells in lung with multicytokine coexpressions. (A & B) Mice were primed and boosted with Rv3615c+CpG I.N. or S.C., and killed at the indicated time intervals. Mononuclear cells from lung, spleen, and LN tissues were isolated. Representative dot plots from flow cytometry analysis in (A) showed the identification of effector–memory CD4^+^ T cells (T_EM_, CD44^+^ CD62^low^). Frequency of the T_EM_ in CD4^+^ T‐cell populations at indicated time intervals was shown in (B) and compared with Student's *t* test. **P < 0.01; ***P < 0.001. (C & D) Mice were primed and boosted with Rv3615c+CpG I.N. or S.C., and killed at week 12 after boosting. Mononuclear cells from lung and spleen tissues were isolated and stimulated with Rv3616c_41‐50_. Brefeldin A was added into the cultures during the final 6 h. The cells were harvested and stained with fluorochrome‐conjugated monoclonal antibodies and analysed for lineage differentiation and intracellular cytokine expression with flow cytometry. Representative dot plots from flow cytometry analysis in (C) showed identification of cytokine‐producing CD4^+^ T cells. Proportion of single‐ and multicytokine‐producing cells in IFN‐γ‐producing population was shown in pie chart (D) and compared with Student's *t* test. Data shown were from seven mice and expressed as mean ± SD

We studied the differentiation of CD4^+^ T cells following I.N. or S.C. immunization with Rv3615c+CpG. Data showed that I.N. immunization induced a higher frequency of CD44^+^CD62L^low^ expressions in lung CD4^+^ T‐cell population than S.C. immunization did (Figure 5A and B). Kinetic data showed that the significant increase of CD44^+^CD62L^low^ cells started from week 6 after the boost, and maintained at least for 16 weeks. Cells in the spleen and LN had similar kinetics and frequency of CD44^+^CD62L^low^ expression following I.N. and S.C. immunizations. Here, we defined cells with CD44^+^CD62L^low^ phenotype as effector–memory cells (T_EM_),^8^ and our data suggested that I.N. immunization preferably increased the frequency of T_EM_ at lung tissue compartment.

We also studied the profile of cytokine productions at week 12 after immunization with Rv3615c+CpG. Here, we used IFN‐γ production as an indicator of cell response to immunization. Our data showed that mice with I.N. immunization had a higher frequency of CD4^+^ T cells in the lungs than did mice with S.C. immunization, coexpressing triple‐ and double‐cytokines, such as IFN‐γ^+^TNF‐α^+^IL‐2^+^ triple expressions (11.8 ± 2.85 vs 5.2 ± 4.51, *P* < 0.01), IFN‐γ^+^IL‐2^+^ double expressions (18.3 ± 5.31 vs 9.4 ± 3.93, *P* < 0.001), and IFN‐γ^+^TNF‐α^+^ double expressions (5.4 ± 1.09 vs 2.9 ± 2.30, *P* < 0.05). But mice with S.C. immunization had a higher frequency of CD4^+^ T cells that expressed IFN‐γ only in the lungs. Cells in the spleen had a similar pattern of cytokine expressions following I.N. and S.C. immunizations (Figure 5C and D). Our data suggested that I.N. immunization promoted a response of cells in lung tissues and induced differentiation towards an effector–memory phenotype with a potency of multicytokine productions.

### 3.5 Intranasal immunization preferably expanded CD4^+^ T cells in BALF and lung parenchyma

**Figure 6:**
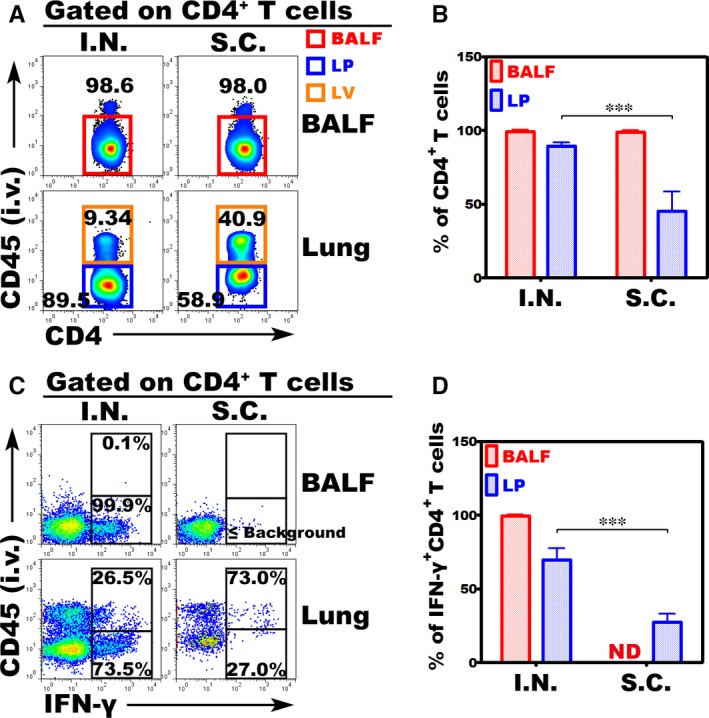
Intranasal and subcutaneous immunization‐expanded CD4^+^ T cells with distinct tissue location. Mice were immunized with Rv3615c+CpG I.N. or S.C., and killed at week 12 after boosting. Fluorochrome‐conjugated anti‐CD45 antibody was injected intravascularly (I.V.) into mice 5 min before killing to label cells in circulation. Mononuclear cells in BALF and lung samples were isolated and suspended in complete RPMI 1640 medium. (A & B) Cells were stained with fluorochrome‐conjugated monoclonal antibodies for lineage differentiation and analysed with flow cytometry. Representative dot plots in (A) showed the distinguishing of cells in compartments of lung (parenchyma, LP; vasculature, LV). Relative proportion of cells in compartments of lung induced by I.N. and S.C. immunization was shown in (B). (C & D) The cells were stimulated with Rv3616c_41‐50_. Brefeldin A was added into the cultures during the final 6 h. The cells were harvested and stained with fluorochrome‐conjugated monoclonal antibodies and analysed for lineage differentiation and intracellular cytokine expression with flow cytometry. Representative dot plots in (C) showed the identification of IFN‐γ‐producing cells in compartments of lung. Relative proportion of IFN‐γ‐producing cells in BALF and LP induced by I.N. and S.C. immunization was shown in (D). Data were from five mice and compared with Student's *t* test. Data were expressed as mean ± SD. ***P < 0.001; ND: not detected (equal or less than background)

We explored the location of cells responding to I.N. and S.C. immunization. Cells in LP and LV were distinguished by in vivo labelling with fluorochrome‐conjugated anti‐CD45 antibody following a reported procedure 37 with minor modifications. Most T cells in BALF (>98%) were not stained by in vivo labelling due to the anatomic location, which demonstrated reliability of our labelling protocol (Figure 6A). We sampled mononuclear cells from lungs of mice at week 12 after immunization. Mice with I.N. immunization had the most CD4^+^ T cells detected in LP (>89%), while mice with S.C. immunization had roughly half in the parenchyma and half in LV (Figure 6A and B). With intracellular cytokine staining, we also found that I.N. immunization induced IFN‐γ‐producing cells that were mostly located in BALF and LP (99.9% and 73.7%, proportion of cells that were CD45^−^ in IFN‐γ^+^ population), while those induced by S.C. immunization were only 22.5% in parenchyma, and hardly to be seen in BALF (Figure 6C and D). Our data suggested that I.N. immunization selectively primed and expanded CD4^+^ T cells in BALF and LP, and increased their proportions in the total lung CD4^+^ T‐cell compartment, while S.C. immunization systemically expanded CD4^+^ T cells mostly in circulation.

### 3.6 Immunization‐expanded CD4^+^ T cell with different tissue locations had distinct profile of phenotype and cytokine expressions

**Figure 7:**
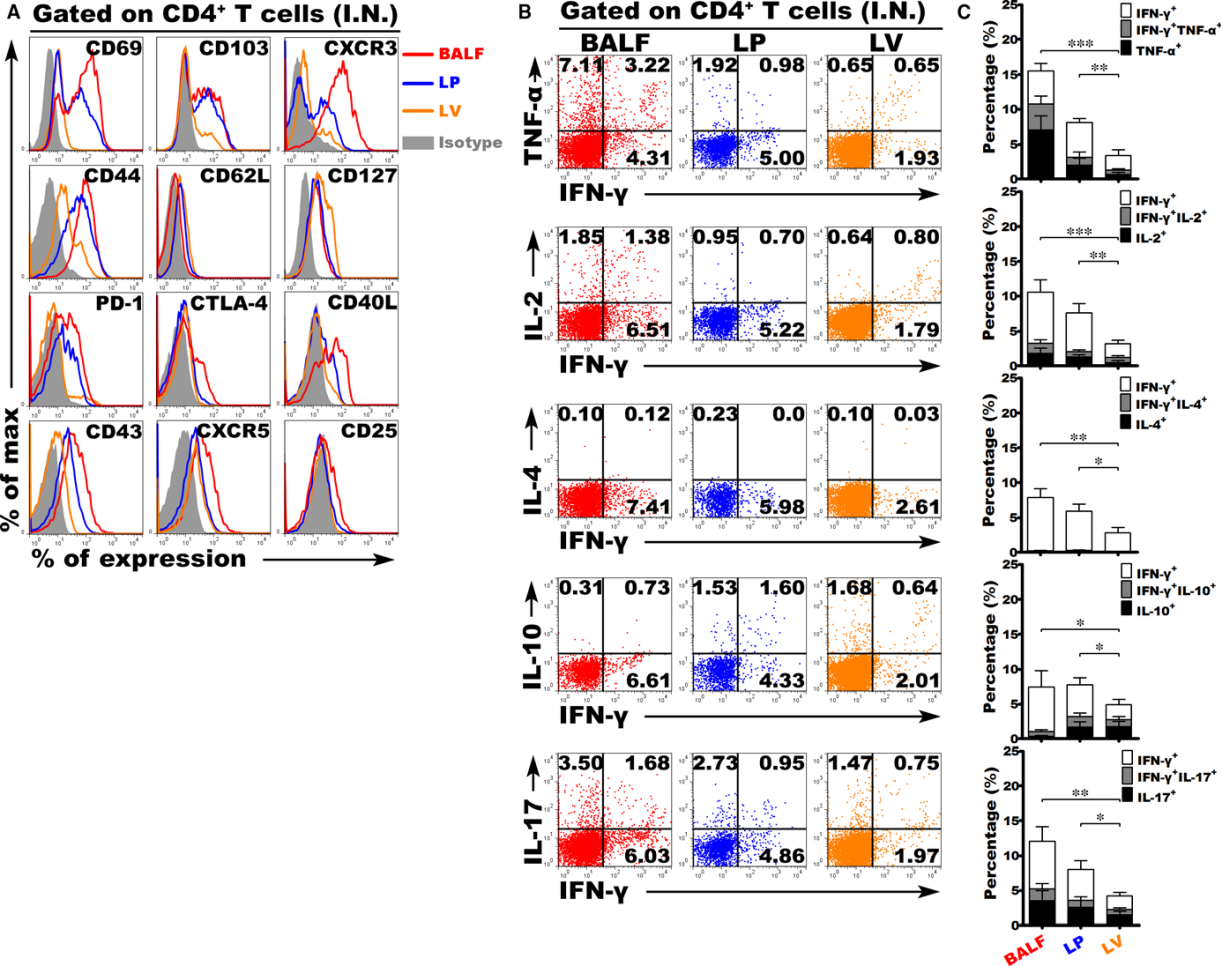
CD4^+^ T cells with distinct localization had different profile of phenotype and cytokine expressions. Mice were immunized with Rv3615c+CpG I.N., and killed at week 12 after boosting. Mononuclear cells from BALF and lung samples were isolated. (A) Cells were stained with fluorochrome‐conjugated monoclonal antibodies for lineage differentiation and indicated phenotypes. Representative histograms showed phenotype expression among CD4^+^ T cells with distinct localization. (B & C) Cells were stimulated with Rv3616c_41‐50_. Brefeldin A was added into the cultures during the final 6 h. The cells were harvested and stained with fluorochrome‐conjugated monoclonal antibodies for lineage differentiation and intracellular cytokine expression. Representative dot plots in (B) showed identification of cytokine‐producing cells with flow cytometry. Proportion of cells with single or multiple cytokine expressions was compared by Student's *t* test (C). Data shown were representative of five mice, and were expressed as mean ± SD. *P < 0.05; **P < 0.01; ***P < 0.001

Immunization‐expanded cells in tissues might have a distinct profile of phenotype and cytokine expressions, representing functional diversity. We studied CD4^+^ T cells isolated from BALF and lung tissues of mice at week 12 of immunization with Rv3615c+CpG. More cells in BALF and LP expressed molecules associated with tissue residency (CD69 and CD130), chemokine receptors (CXCR3 and CXCR5), memory differentiation (CD127), costimulation (PD‐1, CTLA‐4 and CD40L), and activation (CD43 and CD25) than did cells in LV, while more cells in LV expressed molecules associated with homing (CD62L) (Figure 7A, and Table 1). We also studied cytokine profile of cells in tissues. In general, more cells in BALF and LP expressed cytokines compared to those in LV. Among them, IFN‐γ single, IFN‐γ^+^ TNF‐α^+^ double, and IFN‐γ^+^IL‐2^+^ double expressions in BALF and LP were significantly higher. In contrast, more cells in LV expressed IL‐10^+^ single, and IL‐10^+^IFN‐γ^+^ double than those in BALF and LV did (Figure 7B and C). The phenotype and cytokine profiles indicated functional diversity of cells in different tissue compartments responding to immunizations.

**Table 1:** The phenotypes of CD4^+^ T cells in BALFs, lungs parenchyma (LPs), and lung vasculature (LVs)

Marker	BALF	LP	LV
Tissue residence			
CD69	60.9 ± 7.02%	44.6 ± 5.23%	8.27 ± 0.89%
CD103	51.5 ± 6.56%	38.9 ± 4.56%	11.6 ± 1.27%
Chemokine receptor			
CXCR3	79.6 ± 3.89%	45.6 ± 3.89%	17.7 ± 2.36%
CXCR5	70.2 ± 4.70%	62.3 ± 3.10%	44.8 ± 2.10%
Effector–memory			
CD44	9.26 ± 3.47%	11.8 ± 4.53%	13.4 ± 3.26%
CD62L	38.6 ± 0.30%	47.1 ± 0.89%	63.4 ± 2.76%
CD127	25.7 ± 4.44%	16.8 ± 5.11%	8.53 ± 7.89%
Costimulation			
PD‐1	13.2 ± 2.59%	6.66 ± 1.47%	3.84 ± 0.76%
CTLA‐4	38.7 ± 2.20%	16.5 ± 2.17%	7.29 ± 0.50%
CD40L	56.6 ± 5.21%	24.4 ± 1.89%	9.04 ± 0.66%
Activation			
CD43	35.8 ± 4.58%	20.8 ± 2.10%	14.8 ± 1.10%
CD25	8.05 ± 1.20%	3.27 ± 1.69%	1.62 ± 0.23%

## 4 DISCUSSION

Establishment of a long‐term immune memory to a specific pathogen is essential for the success of a vaccination.^38^ In our previous study,^9^ we demonstrated that Rv3615c contained epitopes of human CD4^+^ T cells, and elicited Th1‐type dominant responses of CD4^+^ T cells from patients with tuberculosis pleurisy, which suggested a potential for inducing protective immunity against *M.tb*. To extend our knowledge, here, we used a mouse model to demonstrate that Rv3615c immunization not only elicited a Th1‐type response, but also induced memory differentiation of CD4^+^ T cells. We identified that Rv3615c contained an epitope of mouse CD4^+^ T cells, which is located within amino acids 41‐50 (Rv3615c_41‐50_) and is differed from epitopes of human CD4^+^ T cells, as we have previously reported. The quantity of IFN‐γ and number of IFN‐γ‐producing cells induced by Rv3615c_41‐50_ were the same as those induced by full‐length Rv3615c and mixture of overlapping peptides. Although a few CD4^−^ T cells expressed IFN‐γ, the frequency was low. Thus, we demonstrated the Rv3615c_41‐50_ containing a dominant epitope of mouse CD4^+^ T cells. After prime and boost with Rv3615c, the cytokine‐producing cells peaked at week 4. Most of the cytokine‐producing cells expressed IFN‐γ and TNF‐α; some of them expressed IL‐17 and IL‐2 in the spleen, lung, and LN. A few of them expressed IL‐4, but the IL‐4 production was only transient. The IFN‐γ‐, TNF‐α‐, and IL‐2‐producing cells were maintained for at least 16 weeks, while the IL‐17‐producing cells declined in frequency significantly after 12 weeks. Since antigen‐specific memory CD4^+^ T cells with a Th1‐type profile were associated with a reduced *M.tb* burden in the animal model, an improved bacterial clearance, and less severe pulmonary inflammation in the human clinical trial,^39^, ^40^ our data suggested that Rv3615c induced a sustained adaptive immunity with the potential for a rapid response to *M.tb* challenge and protection against infection.

Immunization‐induced memory cells located at the site of pathogen invasion are crucial for timely and efficient response to an infection. Different immunization routes utilize different subsets of antigen‐presenting cells, and consequently activate different subsets of CD4^+^ T cells at their specific tissue compartment^26^; establishing a long‐term resident CD4^+^ memory T‐cell population in barrier tissues through local immunization promoted regional immunity and demonstrated a key point for vaccine design.^4^, ^41^ S.C. immunization expanded Rv3615c‐specific CD4^+^ T cells systemically, in terms of elevated cytokines in serum, frequency of cytokine‐expressing cells, and quantity of cytokines in the lung, spleen, and LN tissues. Additionally, the cytokine‐expressing cells in the lungs were mostly in the vasculature, indicating a circulatory status. In contrast, I.N. immunization preferably elevated cytokines and cytokine‐producing cells in BALF and LP, suggesting a status of tissue residency. The quantity of cytokines and frequency of cytokine‐producing cells in lung were higher than those in the spleen and LN, and higher than those induced by S.C. immunization. In addition, I.N. immunization promoted not only regional immunity in the respiratory compartment but also systemic immunity, in terms of increased frequency of cytokine‐producing cells and cytokine productions in the spleen and LN at the level comparable to that induced by S.C. immunization. I.N. immunization with Rv3615c also established a sustained adaptive B‐cell immunity in the airway. The elevated Rv3615c‐specific antibodies in sIgA, IgA, IgG1, and IgG2a isotypes were maintained in BALF for at least 8‐12 weeks post the prime and boost, and antibodies in IgM isotype were maintained for at least 6 weeks. I.N. immunization also elevated Rv3615c‐specific sIgA and IgA in serum, while S.C. immunization had no significant impact on antibody levels in BALF but elevated Rv3615c‐specific IgG1 and IgG2a only in serum. Although the role of antibody response on preventing *M.tb* infection and in eradicating the pathogen is still in debate and requires further investigation,^42^, ^43^ our study has demonstrated that I.N. immunization establishes a sustained adaptive immunity in the respiratory tract and in lung tissue, posing an advantageous response to pathogenic invasion without time‐consuming recruitment of effector cells from peripheral lymphoid tissues.^44^

Efficiency of adaptive immunity depends not only on the magnitude of response but also on functional quality of cells responding to the infection. T cells with IFN‐γ only production were characterized as terminally differentiated and exhausted, and associated with active TB progression,^45^, ^46^ while effector–memory CD4^+^ T cells with multicytokine coexpression were associated with control of *M.tb* and other infections.^47^ I.N. immunization showed preference of inducing CD44^+^CD62L^low^ effector–memory CD4^+^ T cells (CD4^+^ T_EM_) in the lung, with a frequency higher than those in the spleen and LN, and those induced by S.C. immunization. Many CD4^+^ T_EM_ cells coexpress multicytokines. The frequency of IFN‐γ^+^ TNF‐α^+^ double‐expressing, and IFN‐γ^+^ TNF‐α^+^ IL‐2^+^ triple‐expressing cells induced by I.N. immunization was significantly higher than those induced by S.C immunization, and higher than the cells in the spleen and LNs induced by the same immunization. The elevated frequency of CD4^+^ T_EM_ and multicytokine coexpressing cells in the lung was maintained for at least 16 and 12 weeks respectively. Multicytokine coexpression and effector–memory phenotype represented a potency of CD4^+^ T cells in eradicating the pathogen and controlling the disease progression.^45^ Our data suggested that I.N. immunization with Rv3615c established an adaptive immunity with cells possessing functional quality and a sustainable response.

Upon further analysis of cells after the prime and boost, our data revealed that I.N. immunization‐expanded cells were mostly in the respiratory tract and LP, while majority of S.C. immunization‐expanded cells were in the LV. Cells in BALF and LP had a higher frequency of effector–memory differentiation (CD62L^low^ and CD127^+^), tissue residency (CD69^+^ and CD103^+^), migration and mucosal tissue homing (CXCR3^+^ and CXCR5^+^), costimulation (PD‐1^+^, CTLA‐4^+^ and CD40L^+^) and activation (CD43^+^ and CD25^+^) phenotypes, and higher frequency of IFN‐γ^+^ and TNF‐α^+^ single and multicytokine (such as IFN‐γ^+^TNF‐α^+^, IFN‐γ^+^IL‐2^+^) expressions than did cells in LV. This is consistent with their property of sustainable and rapid response to antigen stimulation. Studies showed that memory T cells located in mucosal tissues enhanced regional immunity by rapid control of infections.^11^, ^26^, ^41^, ^48^ These tissue‐resident memory T cells formed an immune barrier and functioned as a defence line to pathogenic invasions, and often coexpressed multicytokines and were potent at eliminating invading pathogens.^11^, ^26^ Thus, I.N. immunization with RV3615c could be an effective way to induce adaptive immunity in the airway and the lungs.

In our prime and boost procedure, adjuvant CpG promoted immunogenicity of Rv3615c. The use of CpG was based on our experience and previous studies.^49^ The adjuvant might activate the innate immune system, lower the threshold for signalling of activation, and reduce the quantity of antigen required for initiating an immune response.^36^ We have not, here, tried to figure out the mechanism of CpG's promoting of magnitude and altering of pattern of response to Rv3615c. Also, we have not distinguished if our I.N. prime and boost selectively expanded precursors of effector–memory CD4^+^ T cells in the respiratory tissue compartment or initiated migration and differentiation of naïve cells in peripheral lymphoid tissues, although the magnitude of response was relatively low in the lymphoid tissues. However, as an extension to our previous investigation, we have demonstrated that I.N. immunization with Rv3615c establishes a sustained adaptive immunity and a potential of rapid response to *M.tb* infections in the local respiratory system. The I.N. inoculation is not novel for test of TB and other vaccinations. To our knowledge, there are few, if any, reports on Rv3615c‐induced responses induced by I.N. in mouse model. We believe that our data provide information to support further investigation of the Rv315c as a vaccine candidate delivered by alternative routes. Limited by biosafety regulations, we are not qualified to test if immunization with Rv3615c could protect mice from a virulent *M.tb* challenge, and the potential of inducing sustainable adaptive immunity in humans requires further clinical study. The benefit of inducing effector–memory CD4^+^ T cells with multicytokine production and a tissue‐resident property remain unclear in the context of this mouse model. Since *M.tb* proteins are expressed at different stages of the infection and induce distinct immune responses,^50^ immunization with Rv3615c elicits additional protection beyond those induced by BCG. We propose that a sustained vaccine‐induced CD4^+^ T‐cell response against a broad spectrum of targets, and with diverse functionalities, provides a better protection compared to a narrow response. Our study provides important information related to inducing broad spectrum and quality immunity against *M.tb* infections, and sheds light on the rational design of vaccines.

## CONFLICTS OF INTEREST STATEMENT

The authors confirm that there are no conflicts of interest.

## AUTHORSHIP

Changyou Wu, Jie Liu and Jiangping Li designed research; Jiangping Li, Jun Zhao, and Juan Shen performed research and analysed data; Jiangping Li, Changyou Wu, and Jie Liu wrote the paper; All authors approved the submitted and final versions.
